# Distinct Neural Activity Associated with Focused-Attention Meditation and Loving-Kindness Meditation

**DOI:** 10.1371/journal.pone.0040054

**Published:** 2012-08-15

**Authors:** Tatia M. C. Lee, Mei-Kei Leung, Wai-Kai Hou, Joey C. Y. Tang, Jing Yin, Kwok-Fai So, Chack-Fan Lee, Chetwyn C. H. Chan

**Affiliations:** 1 Laboratory of Neuropsychology, The University of Hong Kong, Hong Kong, China; 2 Laboratory of Cognitive Affective Neuroscience, The University of Hong Kong, Hong Kong, China; 3 The State Key Laboratory of Brain and Cognitive Sciences, The University of Hong Kong, Hong Kong, China; 4 Social Neuroscience Research Network, The University of Hong Kong, Hong Kong, China; 5 Number Laboratory, The University of Hong Kong, Hong Kong, China; 6 Centre of Buddhist Studies, The University of Hong Kong, Hong Kong, China; 7 Department of Anatomy, The University of Hong Kong, Hong Kong, China; 8 Applied Cognitive Neuroscience Laboratory, Department of Rehabilitation Sciences, The Hong Kong Polytechnic University, Hong Kong, China; CNRS - Université Claude Bernard Lyon 1, France

## Abstract

This study examined the dissociable neural effects of *ānāpānasati* (focused-attention meditation, FAM) and *mettā* (loving-kindness meditation, LKM) on BOLD signals during cognitive (continuous performance test, CPT) and affective (emotion-processing task, EPT, in which participants viewed affective pictures) processing. Twenty-two male Chinese expert meditators (11 FAM experts, 11 LKM experts) and 22 male Chinese novice meditators (11 FAM novices, 11 LKM novices) had their brain activity monitored by a 3T MRI scanner while performing the cognitive and affective tasks in both meditation and baseline states. We examined the interaction between state (meditation vs. baseline) and expertise (expert vs. novice) separately during LKM and FAM, using a conjunction approach to reveal common regions sensitive to the expert meditative state. Additionally, exclusive masking techniques revealed distinct interactions between state and group during LKM and FAM. Specifically, we demonstrated that the practice of FAM was associated with expertise-related behavioral improvements and neural activation differences in attention task performance. However, the effect of state LKM meditation did not carry over to attention task performance. On the other hand, both FAM and LKM practice appeared to affect the neural responses to affective pictures. For viewing sad faces, the regions activated for FAM practitioners were consistent with attention-related processing; whereas responses of LKM experts to sad pictures were more in line with differentiating emotional contagion from compassion/emotional regulation processes. Our findings provide the first report of distinct neural activity associated with forms of meditation during sustained attention and emotion processing.

## Introduction

It has been widely speculated that longterm meditation training has a significant positive impact on neuropsychological functioning in both cognitive and affective domains [1], [2]. Here we report a study on the dissociable effects on neural activity of two forms of meditation following the Theravāda school of Buddhism: *ānāpānasati* (focused-attention meditation) and *mettā* (loving-kindness meditation).

Among the broad array of meditation practices, the most basic and widely studied form is concentrative or focused-attention meditation (FAM). FAM practitioners focus their entire attention upon an object or a bodily sensation and, whenever they are distracted by external stimuli or inner thoughts, they bring their attention back to that object or sensation. The goal is to achieve a clear (vivid) and unwavering (calm and stable) state free from distraction. FAM's reported major longterm benefit is cognitive—attentional control. For example, expert meditators show larger mismatch negativity amplitudes, a measure of attention. Mismatch negativity is an event-related potential waveform reflecting the involuntary attentional switching that can be elicited by the appearance of an infrequent stimulus in a stream of frequent stimuli. The larger mismatch negativity amplitudes observed in the experts (with 3 to 7 years of daily practice) implies their higher ability, relative to the matched novice meditators, in detecting changes appeared in the auditory task—especially after meditation—suggesting that they are more efficient at preattentive detection of signal changes [3].

The relationship between the strength of BOLD signals and meditation experience appears to follow an inverted u-shaped function. Compared to novices, experts with at least 3 years of experience actually had lower sustained activation in attention-related brain areas, including the left superior frontal gyrus (SFG) and cingulate cortex [4]. However, Buddhist meditators with an average of 7.9 years of meditative practice (equivalent to 5,767 hours, 2 hours daily) showed higher activation in the medial prefrontal cortex (mPFC) and the anterior cingulate cortex (ACC) during *Vipassana* (Buddhist mindfulness meditation) compared to matched controls who had no previous experience with meditation or similar practices [5]. Also, experts with 10,000–24,000 hours of practice (mean 19,000 hours) showed significantly more activation in attention-related regions, compared to age-matched novices who had no prior experience except in the week before the experiment [6]. On the other hand, longterm practitioners who averaged more than twice as much meditation experience (mean 44,000 hours) showed less activation in attention-related regions. The authors interpreted this inverted u-shaped function to reflect skill acquisition—a pattern that has been observed in others domains of expertise [7], [8]. The findings are consistent with meditation texts that describe concentration meditation as initially requiring greater levels of effort but later becoming less effortful. So experts seem to “settle” into meditative states with minimal effort.

There has been a recent surge of research interest in the effect of loving-kindness meditation (LKM) on brain functioning. LKM emphasizes a state of universal love and compassion, equalizing the self and others [9], [10]. Compassion cultivates the desire to relieve pain and suffering for the self and others, while loving-kindness loads the mind with universal, nonreferential compassion towards oneself and other beings [11]. Practitioners of LKM imagine a being—human or animal—and flow unconditional love and benevolence towards that being; they extend this love to all sentient beings and wish that all living beings are free from suffering and its causes. Love and compassion eventually grow and fill the entire mind, with no other consideration, reasoning, or discursive thoughts. Disparate as they may seem, LKM and FAM can be complimentary: a focused state enables people to sustain universal, nonreferential love and compassion; conversely, the feeling of love and kindness helps people achieve a peace of mind useful for entering into a focused state [10].

Compared to FAM, relatively little is known about the neural basis of LKM. Lutz and colleagues [11] played LKM experts emotional sounds during meditation and baseline and found that they had increased neural activity during meditation including the anterior insula, postcentral gyrus, inferior parietal lobule (IPL), amygdala, right temporal-parietal junction, and right posterior and superior temporal sulcus. They interpreted the findings as meaning that LKM experts have a higher level of integration of sensory-perceptual processes and affective responses than novices. In a follow-up study, Lutz and colleagues [12] further confirmed that LKM experts—relative to novices—had more activity in the left somatosensory cortex, IPL, ACC, and insula in response to emotional sounds. These findings suggest that longterm LKM practice may enhance sensitivity to the emotional experiences of others, which may be similar to empathy [13]. Thus far no study has directly compared neural activity measured by BOLD signals associated with FAM and LKM during cognitive and affective tasks. This study fills this research gap by investigating the overlapping and distinct neural correlates of FAM and LKM with cognitive and affective processing. Cognitive performance with the continuous performance test (CPT) and affective processing with the emotion-processing task (EPT), which involves viewing happy, sad, and neutral photos from the International Affective Picture System (IAPS), were employed as the experimental tasks. For each task, two voxel-wise analyses using a 2×2 factorial design were conducted for the two forms of meditation respectively, with state (meditation and baseline) as a within-subject factor and group (experts and novices) as a between-subjects factor. We hypothesized that the neural activity associated with different states and groups during the CPT and the EPT would be distinct between FAM and LKM. To test this hypothesis, we examined the overlapping and distinct neural activity.

Since attention is the training goal of FAM, we predicted that there would be differences in BOLD signals in attention-related brain areas during the CPT, including the lateral PFC, IPL, superior temporal gyrus (STG), cingulate cortex, and caudate, regions that has previously been reported to have connected to attention [4], [14]–[16]. We predicted that FAM experts would have stronger signals in these regions during meditation versus baseline and that this effect would be even stronger for FAM experts over novices. On the other hand, because LKM focuses on emotional training, we predicted that LKM mediators would have stronger BOLD signals in emotion-related brain areas while they viewed affective pictures. Those areas include the PFC (dorsomedial PFC, lateral PFC, and orbitofrontal cortex), parietal and temporal cortical regions, and limbic regions, including the insula, cingulate regions, amygdala, hippocampal region, and the caudate [17]–[21]. We hypothesized that LKM experts would have stronger signals in these regions during meditation versus baseline and that this effect would be stronger for LKM experts than novices. We also sought to verify whether or not activity in these brain regions correlated with performance on the CPT and ratings of the IAPS. Self-reported affect measures were administered in order to verify if our LKM experts, with their years of LKM practice, were better able to reduce negative affect, relative to novices who had only one week of LKM experience.

## Materials and Methods

### Participants

After approval from The University of Hong Kong's Ethics Committee, we recruited experts from a Buddhist meditation network in Hong Kong. Meditators were included if they were male and had practiced FAM or LKM for 2 hours each day for at least 5 years. Exclusion criteria were: history of traumatic brain injury, medical conditions, or any psychiatric disorder that could affect neural activity and brain functioning. A total of 22 Chinese experts were recruited, 11 for FAM and 11 for LKM. They had been practicing either FAM (*ānāpānasati*) or LKM (*mettā*) based on the Theravāda tradition, the oldest Buddhist practice. Theravāda is still prevalent in India, Sri Lanka, and other Southeast Asian countries. The forms of meditation reported by the experts were verified by the teacher of these meditators as well as by Venerable Jing Yin. The FAM experts ranged in age from 39 to 68 years (mean = 52.72±9.69 years), with an average of 14.09±3.21 years of education. The LKM experts ranged in age from 31 to 68 years (mean = 51.82±11.28 years), with an average of 14.27±3.95 years of education. Participants reported having at least 5 years of practice of FAM (mean = 5,248.95±6,191.94 hours; range = 810 to 17,850 hours) or LKM (mean = 7,491.98±6,681.43 hours; range = 588 to 17,850 hours). All practitioners commenced meditation practice in FAM first and then afterwards they chose the form of practice that they wanted to pursue, which in this study was either LKM or FAM. Hence, it was impossible to recruit participants who solely practiced LKM. Nevertheless, since this study examined the state effects of FAM and LKM, the fact that our two groups of meditators had clearly demonstrated expertise in their respective forms of meditation, FAM or LKM, was considered sufficient for verifying the different state effects of the two forms of meditation examined in this study.

Matched healthy volunteers were recruited from the community. Inclusion criteria were: male ages 30 to 65, Chinese ethnicity, interested in meditative training, and no prior meditative practice. A total of 22 novices were recruited and randomly assigned as controls (FAM novices; n = 11) of the FAM experts or the controls (LKM novices; n = 11) of the LKM experts. They were given written instructions for a 1-week, home-based meditation practice, based on their group membership. The Chinese instructions for the home practice of FAM and LKM were designed by one of our co-authors, Venerable Jing Yin, who himself has more than 40 years of experience in meditation. These instructions are very similar to those offered by Venerable Dr. M. Ricard, who has much experience practicing and teaching meditation. The participants were instructed to perform the home practice for 1 hour per day for 7 days consecutively. Specifically, they were asked to separate the 1-hour practice into three 20-minute sessions in order to achieve better effects. After the 1-week practice, the experimenter solicited from the novices self-reports of changes in their meditation experience. The experimenter then sought confirmation from Venerable Jing Yin that the novices were on the right track. The FAM novices ranged in age from 31 to 63 years (mean = 47.16±9.67), with an average of 18.45±2.11 years of education. The LKM novices ranged in age from 36 to 59 years (mean = 47.34±8.95), with an average of 16.73±5.10 years of education. There were no significant differences in terms of age between experts and novices *F*(1, 40) = 2.81, *p*>.10. All participants (experts and novices) were right-handed according to the Edinburgh Handedness Inventory [22] and with at least high school education.

All participants also completed two mindfulness questionnaires, namely the Toronto Mindfulness Scale (TMS) [23] and Cognitive and Affective Mindfulness Scale Revised (CAMSR) [24], prior to the study (i.e. after the 1-week practice for novices). For both scores, no significant group by form-of-meditation interactions [TMS: *F*(1,40) = 0.67, *p*>.1; CAMSR: *F*(1,40) = 0.28, *p*>.5] or main effects for form-of-meditation [TMS: *F*(1,40) = 2.75, *p*>.1; CAMSR: *F*(1,40) = 2.10, *p*>.1] were detected. Only the main effects for group were significant [TMS: *F*(1,40) = 14.49, *p*<.0005; CAMSR: *F*(1,40) = 13.49, *p*<.001], indicating that experts had in general much higher mindfulness scores than novices prior to the study.

### fMRI Tasks

In the CPT, numbers zero to nine were displayed in white, 18-point Arial font for 50 milliseconds (ms) at the center of a dark background once every second. The grayness of the numbers varied randomly on 5 levels. In the experimental condition, participants pressed a button once if the number zero appeared, which happened on one-third of the 450 total trials. Performance is summarized into four scores: commission errors, omission errors, reaction time, and its variability. In the control condition, stimulus presentation was identical, except that participants were instructed to simply look at the screen without attending to or pressing the button for the number zero. The control condition was used to account for the visual components of watching flashing numbers; it represents baseline attention level. All stimuli were generated by E-Prime on a control computer located outside the MR room and displayed in the room using a back projection screen.

**Figure 1:**
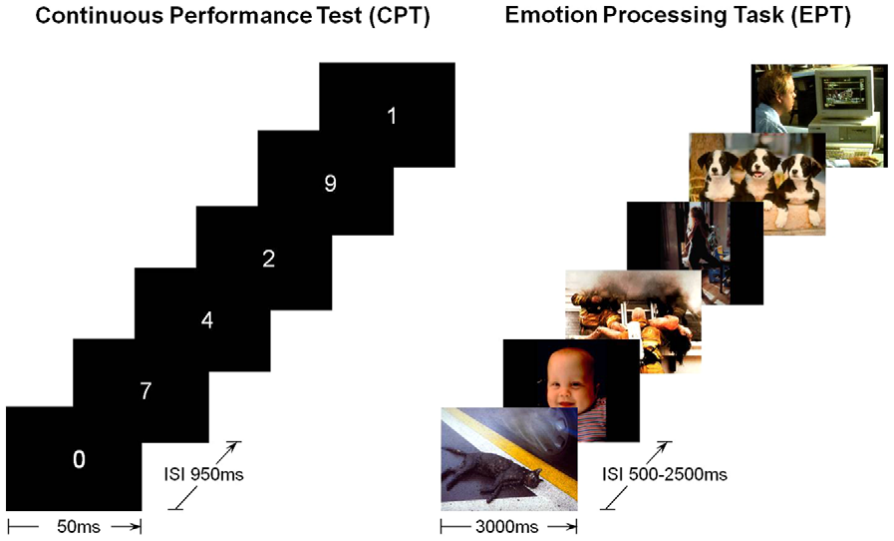
Schematic diagrams of the fMRI experimental tasks.

The EPT included 20 happy, 20 sad, and 20 neutral pictures from the IAPS with the highest valence and arousal ratings in published norms [25]. Each emotion valence had equal proportions of pictures with human and nonhuman images (i.e., animals, objects, and scenes). All stimuli appeared once on a dark background randomly in two 30-trial runs. Each trial had a 3-second stimulus presentation, separated by a white central fixation cross with varying durations from 500 to 2,500 ms in steps of 500 ms (i.e., 500; 1,000; 1,500; 2,000; and 2,500 ms). Participants also rated the valence from 1 (*very negative*) to 9 (*very positive*) and arousal (1 = *not arousing*, 9 = *very arousing*) of each happy and sad picture. Figure 1 illustrates the CPT and EPT. All behavioral data of the CPT and the EPT were analyzed using Statistical Package for the Social Sciences (SPSS, v.16).

### Self-Report Measures

The 20-item Chinese Affect Scale (CAS [26]) assessed positive affect (CAS-PA) and negative affect (CAS-NA). The CAS is culturally adapted to be linguistically and structurally equivalent to the Positive and Negative Affect Schedule (PANAS) [27]. Participants rated the frequency of 10 positive and 10 negative affective states in the previous 2 weeks on a 5-point scale (0 = *not at all*, 1 = *rarely*, 2 = *sometimes*, 3 = *often*, 4 = *all the time*). Separate summation scores were calculated for PA and NA (range = 0–40). The reliability of the CAS has been demonstrated in Chinese young and middle-aged adults (α>.85) [26]. In the current study, the alphas of the CAS-PA and CAS-NA were .87 and .96, respectively. The CAS scores were analyzed using SPSS.

### Experimental Procedures

After being fully informed about the study, participants gave their written informed consent and then completed the self-report measures in a face-to-face interview. Prior to scanning, they were given about 30 minutes to practice FAM or LKM. It was considered that this amount of time would be sufficient for them to enter the meditation state. All the participants then completed the CPT and the EPT during a meditation session (FAM/LKM) and then again during a baseline session (Baseline) separated by a 15-minute break. Both tasks were conducted during scanning. Task order was counterbalanced across participants. After each scan, all participants were asked to rate the clarity (1 = *very unclear*, 9 = *very clear*) and stability (1 = *very unstable*, 9 = *very stable*) of their mental state during the task.

### fMRI Data Acquisition

Whole-brain axial scanning was performed with a 3.0 Tesla Philips Medical Systems Achieva scanner equipped with an 8-channel SENSE head coil. The imaging session involved two acquisitions (FAM/LKM, Baseline) of a series of functional images. Thirty-two functional slices were acquired using a T2*-weighted gradient echo planar imaging sequence (slice thickness = 4 mm, TR = 1,800 ms, TE = 30 ms, flip angle = 90°, matrix = 64×64, field of view [FOV] = 230×230×128 mm, voxel size = 3.59×3.59×4 mm^3^). The axial slices were adjusted to be parallel to the AC-PC plane. The first 6 volumes were discarded to allow for T1 equilibration effects. The acquisitions of FAM/LKM functional scans and Baseline functional scans were separated by a T1-weighted high-resolution anatomical scan (MPRAGE, 164 sagittal slices; TR = 7 ms, TE = 3.2 ms, flip angle = 8°, matrix = 256×240, FOV = 256×240×164 mm; voxel size = 1 mm^3^).

### fMRI Data Analysis

#### Pre-processing

The fMRI data were preprocessed and analyzed using Statistical Parametric Mapping (SPM5; Wellcome Department of Cognitive Neurology, London, UK) in MATLAB 7.7 (Mathworks Inc., Natick, MA, USA). The functional scans were spatially realigned to adjust for head movement and corrected for slice-acquisition timing. Each functional scan was then registered with the anatomical image, warped to the Montreal Neurological Institute (MNI) brain template using a 12-parameter affine transformation and spatially smoothed with an isotropic 8 mm full width at half maximum (FWHM) Gaussian filter. Motion parameters for each session were saved and subsequently included as covariates in the generalized linear model (GLM) in the first-level analyses.

#### First-level single-subject analysis

For the CPT, first-level, single-subject analyses were conducted to derive parameter estimates of four types of block-related activity at each voxel within the brain for the two experimental conditions (FAM/LKM-exp, Baseline-exp) and the two control conditions (FAM/LKM-control, Baseline-control). The control conditions were subtracted from the corresponding experimental conditions (i.e., FAM/LKM-exp minus FAM/LKM-control; Baseline-exp minus Baseline-control) by applying appropriate linear contrasts to the parameter estimates of each block to remove the neural activity for basic visual processing. As a result, two sets of contrast images reflecting only the neural correlates of sustained attention were obtained for both meditation (FAM/LKM) and Baseline states in each subject.

For the EPT, first-level, single-subject analyses were conducted using an event-related model, with six trial types derived respectively for happy, sad, and neutral pictures in FAM/LKM and Baseline sessions: FAM/LKM-hap, Baseline-hap; FAM/LKM-sad, Baseline-sad; FAM/LKM-neu, Baseline-neu. The reason for separating the trials of happy and sad pictures is that the processing of happy and sad emotions may involve different neural networks [21]. The onset of each trial was modeled with a canonical hemodynamic response function, becoming a neural event that represented the association between neuronal activation and blood-flow changes. Each trial type included separate regressors, which served as parameter estimates for the average hemodynamic responses evoked in each trial. These models were used to construct a set of within-subject contrast images to represent the estimated amplitude of the hemodynamic responses for viewing happy, sad, and neutral pictures. The experimental conditions of EPT were the trials viewing happy (FAM/LKM-hap, Baseline-hap) and sad pictures (FAM/LKM-sad, Baseline-sad), whereas the trials viewing neutral pictures represented the baseline condition (FAM/LKM-neu, Baseline-neu). Similarly, using the subtraction method used with the CPT (e.g., FAM-hap minus FAM-neu, Baseline-sad minus Baseline-neu, and so on), four sets of contrast images reflecting the neural correlates of happy and sad emotion processing were obtained for both meditation (FAM/LKM) and Baseline states in each subject.

#### Second-level group analysis: One-sample *t*-test

The validity of the fMRI paradigms (the CPT and the EPT) was confirmed by comparing regions of activation observed in this study with those reported in previous neuroimaging studies on attention (captured by the CPT) and emotion processing. The contrast images of all novices when they were performing the task during baseline (Baseline-exp minus Baseline-control) were grouped for one-sample *t*-tests, with the threshold at *p*<.001 (uncorrected) and a cluster extent of 10 contiguous voxels (120 mm^3^, unless otherwise specified). This combination of *p* value and extent thresholding reduced the effective per-voxel false positive rate to a corrected *p*<.05 [28].

#### Second-level group analysis: Analysis of variance

The contrast images of each subject were entered into second-level, voxel-wise, two-way ANOVA tests (2×2 factorial design), with state (meditation and baseline) as a within-subject factor, and group (experts and novices) as a between-subjects factor. For each task (CPT, EPT-hap, and EPT-sad), one ANOVA model was constructed for one form of meditation practice (FAM and LKM). As a result, 6 two-way ANOVA models were built in all. Interaction effects—obtained by applying appropriate linear contrasts—are reported and discussed here to identify the distinct neural activity associated with different states and groups when performing the CPT and the EPT. The main effects of state were reported in the supporting information (Table S1). The statistical maps (SPM [*F*]) generated respectively were then thresholded at *p*<.001 (uncorrected) with a cluster extent of 10 contiguous voxels.

A series of *post hoc* pairwise *t*-contrasts were conducted to identify the levels of the two factors at which the effects detected by the *F*-contrasts (if any) could be explained. All the *t*-statistical maps were also thresholded at *p*<.001 (uncorrected) with a cluster extent of 10 contiguous voxels.

#### Conjunction and exclusive masking analyses

To address the *a priori* hypothesis of dissociability between the task-related activation patterns in FAM and LKM, conjunction and exclusive masking techniques were used to identify significant activations that were common and unique to FAM and LKM. For conjunction analysis, the interaction effects for FAM and LKM were thresholded at *p*<.001 (uncorrected) and then saved as two individual image outputs (i1 for FAM and i2 for LKM). They were then tested with a logical AND statement [“(i1>0) & (i2>0)”] in the image calculator (ImCalc) implemented in SPM to examine for neuronal overlap between FAM and LKM (with “conjunction null” as the null hypothesis). For exclusive masking analysis, one interaction effect was saved at *p*<.001 (uncorrected), while the effect to be used as the mask was saved at *p*<.05 (uncorrected). Then, other logical statements [“(i1>0)>(i2>0)” when i2 was the mask; “(i2>0)>(i1>0)” when i1 was the mask] were used to look for neuronal distinction between FAM and LKM. The final image outputs resulting from both analyses were thresholded with a cluster extent of 10 contiguous voxels, so that all the resultant clusters were greater than 10 voxels. The resultant images were overlaid onto a high-resolution anatomical image in MNI space (Colin27_T1_seg_MNI.nii; courtesy of Simon Eickhoff) to identify the anatomical name of the results, with frequent consultation of anatomical atlases.

#### Region-of-interest analysis

To further explore the underlying pattern of neural activity behind the significant interaction effect(s) found from the above analysis (if any), mean percent signal change in each of these suprathreshold clusters was examined across different conditions. The signal change data was extracted using region-of-interest (ROI) analysis with the MarsBar toolbox (release 0.42, http://marsbar.sourceforge.net/) [29]. To do so, the voxels in a suprathreshold cluster were used to build an ROI mask, and the anatomical confines of the reported brain region for that suprathreshold cluster were used to modify the ROI mask (in other words, the significantly activated voxels that were spatially outside the anatomical brain region were not included in the ROI mask). This allowed us to examine only the evoked activity that was anatomically sensible and plausible, which should minimize the problems of imperfect voxel-wise correspondence across different individuals. The mean percent signal changes were then obtained by averaging the signal changes within the modified ROI masks for the corresponding conditions in each subject. After that, the net mean percent signal changes representing the neural activity of sustained attention in CPT and emotion processing in EPT were obtained by subtracting the mean percent signal changes in the baseline condition from the experimental condition. Hence, the same 2×2 factorial analyses were conducted on the net mean percent signal change data in SPSS to further delineate the interaction effects. A series of post-hoc (paired and independent) *t*-tests were then conducted.

#### Correlation analysis

We used correlations to measure the relationship between behavioral performance and its respective neural activity (measured by the percent signal change) in the experts. By considering the signals in all voxels in the modified ROI masks, we could avoid the “nonindependence error” of fMRI correlations [30]. Hence, we could get an unbiased measure of the association between evoked activity and individual difference(s) in behavioral performance. CPT performance included commission errors, omission errors, reaction time, and its variability. For the EPT, we examined the correlations of brain activity with ratings of valence and arousal.

## Results

### Behavioral results

Table 1 summarizes all of the behavioral results: performance on the CPT, ratings of IAPS pictures, and self-report measures of affect. Although there were no significant state-by-group interactions in all four behavioral measures of CPT for both the FAM and LKM practitioners, there were some significant and trend-level main effects. For the FAM practitioners, the main effect of group difference (FAM experts vs. novices) on omission errors was significant [*F*(1,40) = 12.5, *p*<.005], while the main effects of group difference on commission errors [*F*(1,40) = 3.22, *p*<.1] and variability of RT [*F*(1,40) = 3.78, *p*<.1] both reached trend-level significances. For the LKM practitioners, only the main effect of group difference (LKM experts vs. novices) on omission errors was significant [*F*(1,40) = 9.68, *p*<.005]. Since there were no significant differences in omission errors (or other performance measures) between the two groups of novices for FAM and LKM in both states (data not shown), we can rule out the possibility that the FAM novices were just particularly weak on the CPT. Furthermore, the FAM experts seemed to make fewer commission errors than the FAM novices during the meditation (p = .062) but not the baseline state (p = .721). This means that the FAM experts may be better able to withhold making responses to non-target stimuli during meditation.

**Table 1:** Descriptive statistics of all behavioral tasks.

	Focused-Attention Meditation			Loving-Kindness Meditation		
	Experts	Novices	*p*-value	Experts	Novices	*p*-value
	Mean (SD)	Mean (SD)		Mean (SD)	Mean (SD)	
*a. CPT (Meditation state)*						
Commission errors (%)	0.7 (0.7)	2.0 (1.8)	.062	4.7 (11.5)	2.0 (2.9)	.458
Omission errors (%)	2.4 (4.4)	13.8 (11.7)	.010*	3.1 (3.8)	13.0 (15.1)	.059
Reaction time (RT) (ms)	620.5 (68.7)	637 (50.3)	.544	626.1 (62.6)	634 (64.1)	.777
Variability of RT (ms)	65.6 (19.6)	82.3 (25.6)	.125	72.4 (28.4)	73.9 (17.3)	.883
*b. CPT (Baseline state)*						
Commission errors (%)	1.0 (1.1)	1.2 (1.1)	.721	4.1 (9.1)	1.8 (2.2)	.435
Omission errors (%)	3.7 (4.4)	11.6 (10.0)	.032*	3.9 (3.8)	13.8 (15.8)	.068
Reaction time (RT) (ms)	625 (64.3)	625.9 (54.0)	.974	626.5 (70.4)	619.8 (60.0)	.817
Variability of RT (ms)	66.3 (20.9)	78.6 (25.7)	.266	66.8 (23.0)	64.2 (18.7)	.777
*c. Ratings of IAPS pictures*						
Happy: valence	6.8 (1.0)	6.8 (0.8)	.991	6.8 (1.0)	6.6 (0.9)	.568
Happy: arousal	6.3 (1.1)	5.6 (0.6)	.076	6.2 (1.2)	6.0 (0.7)	.585
Sad: valence	3.0 (0.6)	2.7 (0.7)	.251	3.1 (0.7)	2.9 (0.8)	.727
Sad: arousal	6.9 (0.9)	6.4 (0.7)	.192	6.7 (1.1)	6.2 (0.8)	.243
*d. Chinese Affect Scale*						
Positive affect	23.5 (5.2)	21.3 (3.7)	.253	23.5 (5.6)	23.1 (5.5)	.850
Negative affect	11.8 (10.0)	11.0 (3.3)	.799	7.4 (5.2)	15.3 (7.1)	.008**

**Note:** The *p*-value represents the significance of group differences between experts and novices of FAM and LKM using independent-samples *t*-tests. (a–b): For the CPT, commission errors were measured as the percentage of trials that participants still responded on when the target stimulus was not present. Omission errors were measured as the percentage of trials that participants did not respond on when the target stimulus was present. Reaction time (RT) is the amount of time that participants took to press the button after the presentation of target stimulus (for trials that they should respond to and did respond). The variability of RT was measured by its standard deviation. Only the omission errors of FAM experts were significantly fewer than those of FAM novices in both meditation and baseline states (*p<.05, two-tailed). (c): Ratings of valence and arousal of happy and sad pictures adopted from the International Affective Picture System (IAPS). (d): Positive and negative affect were measured by the Chinese Affect Scale. Only the negative affect of LKM experts was significantly lower than that of LKM novices (**p<.01, two-tailed).

There was no significant group difference between experts and novices in the ratings of valence and arousal of happy and sad pictures (Table 1c) and positive affect (Table 1d) for both forms of meditation. LKM experts had significantly lower negative affect than LKM novices *t*(22) = −2.97, *p*<.01. This was not the case between FAM experts and novices (Table 1d).

### Neuroimaging results

#### Validity of the experimental paradigms

The activation maps of the CPT and EPT in our novices resembled the findings of previous studies. In the CPT experimental condition (compared to the control condition), novices had stronger BOLD signals in the right middle frontal gyrus (MFG), left IPL, bilateral STG, bilateral middle temporal gyrus (MTG), right inferior temporal gyrus, and left middle occipital gyrus (MOG; see Figure S1a).

While viewing happy pictures, novices had significant activity in the right ACC, bilateral posterior cingulate cortex (PCC), right insula, right MTG, right precuneus, left caudate, left IPL, left MOG, and visual cortex (the bilateral cuneus; see Figure S1b). While viewing sad pictures, novices had significant BOLD signals in the left SFG, left MOG, right MTG, right thalamus, right ACC, and visual cortex (the left cuneus; see Figure S1c).

#### Whole-brain voxel-wise ANOVA

**Figure 2:**
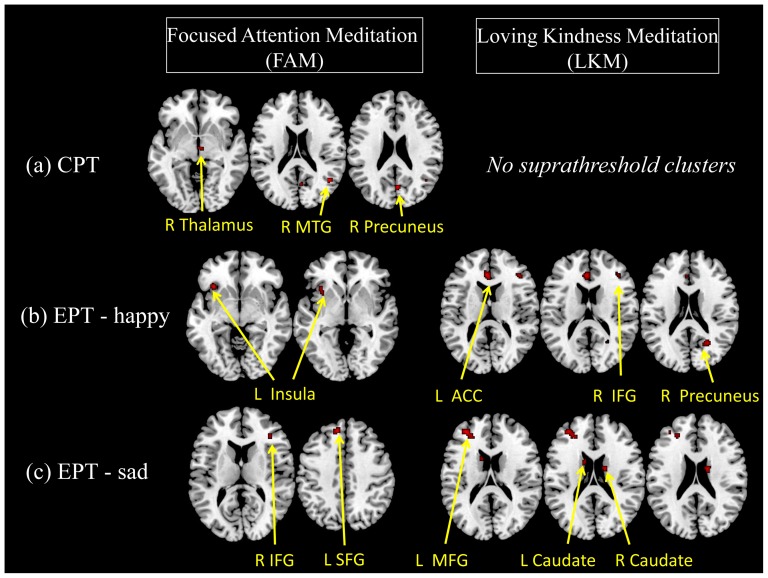
State-by-group interaction of the whole-brain voxel-wise ANOVA. **Notes:** The state-by-group interaction was thresholded at *p*<.001 with a cluster extent of 10 contiguous voxels. L is left, and R is right. All subjects (group: experts and novices) completed the (a) continuous performance task (CPT) and emotion-processing test (EPT), which was subdivided into two parts including viewing of (b) happy pictures (EPT-happy) and (c) sad pictures (EPT-sad) during meditation and baseline states.

On the CPT, there was a significant state-by-group interaction (*p*<.001, k = 10) in the right MTG, thalamus, and precuneus for FAM, but not for LKM (Figure 2a, Table 2a). *Post hoc t*-tests showed that FAM experts had weaker BOLD signals than the FAM novices during baseline in the right thalamus and stronger BOLD signals during meditation in the right MTG and the right precuneus.

**Table 2:** State-by-group interaction of the whole-brain voxel-wise ANOVA.

Task	Meditation	Brain region (Brodmann Area)	Coordinates			*F* value	Cluster size	*t* value			
			x	y	z			Meditation	Baseline	Expert	Novice
(a) CPT	FAM	R Middle Temporal Gyrus (39)	44	−58	20	18.79	14	X	4.40^d^	X	X
		R Thalamus	2	−12	−4	17.6	14	5.99^c^	X	X	X
		R Precuneus (7)	4	−66	24	15.44	29	3.09^c^	X	X	X
	LKM	*no suprathreshold voxels*									
(b) EPT-happy	FAM	L Insula (13)	−34	22	−6	23.99	35	X	X	4.14^e^	X
			−32	14	−2	14.78					
	LKM	L ventral Anterior Cingulate Cortex (24)	−4	34	16	27.23	57	4.40^c^	X	5.17^e^	X
		R Precuneus (7)	26	−60	20	21.7	39	X	X	4.40^e^	X
		R Inferior Frontal Gyrus (45)	42	34	14	20.88	18	4.37^c^	X	X	X
(c) EPT-sad	FAM	L Superior Frontal Gyrus (9)	−10	40	42	25.2	28	X	5.03^c^	X	X
		R Inferior Frontal Gyrus (45)	38	34	12	17.31	11	X	X	X	5.17^e^
	LKM	L Middle Frontal Gyrus (46)	−34	46	18	23.1	86	4.32^c^	X	X	X
			−26	40	20	16.5					
		R Caudate	14	−4	22	18.01	19	X	X	X	4.37^f^
		L Caudate	−12	6	20	16.44	11	4.03^c^	X	X	X

**Note**: The state-by-group interaction was thresholded at *p*<.001 with a cluster extent of 10 contiguous voxels, with state (meditation and baseline) as a within-subject factor and group (experts and novices) as a between-subjects factor. FAM is focused-attention meditation, and LKM is loving-kindness meditation. L is left, and R is right. (a) CPT: continuous performance task, (b) EPT-happy: viewing happy pictures in the emotion-processing task (EPT), and (c) EPT-sad: viewing sad pictures in the EPT. X denotes non-significant.

c expert>novice,

d expert<novice,

e meditation>baseline,

f meditation<baseline.

While viewing happy pictures, there was a significant state-by-group interaction (*p*<.001, k = 10) in the left insula only for FAM and in the left ventral ACC, right inferior frontal gyrus (IFG), and right precuneus for LKM (Figure 2b, Table 2b). *Post hoc t*-tests revealed stronger BOLD signals in the left insula in the FAM experts during meditation than baseline. For the LKM, *post hoc t*-tests showed stronger activity in the experts during meditation than baseline in the left ventral ACC and right precuneus, and stronger activity in the experts than novices during meditation in the left ventral ACC and right IFG.

While viewing sad pictures, the FAM had a significant state-by-group interaction (*p*<.001, k = 10) in the left SFG and right IFG, and the LKM had a significant interaction in the left MFG and bilateral caudate (Figure 2c, Table 2c). For FAM, *post hoc t*-tests revealed stronger activity in the left SFG in both states of experts than the baseline state of novices and stronger activity in the right IFG in the novices during meditation compared to baseline. For LKM, *post hoc t*-tests showed stronger BOLD signals in the experts than novices during meditation in the left MFG and left caudate, and stronger BOLD signals in the novices during baseline compared to meditation in the right caudate [see Fig. S2 for results of the 3-way (state×group×form-of-meditation) interaction analysis].

#### Conjunction analysis

Conjunction analysis did not reveal any neuronal overlap between the state-by-group interactions of FAM and LKM for any of the three tasks (CPT or viewing happy and sad pictures in EPT). The same was true even if a more lenient threshold (*p*<.005) was initially applied to the interaction effects (i.e., when saving i1 and i2 as individual image outputs). In other words, the significant differences between FAM experts and novices in their meditation and baseline states were neither identical nor similar to that between LKM experts and novices with respect to their meditation and baseline states. Therefore, the two forms of meditation—FAM and LKM—do not share the same neural mechanism for attention and emotion processing.

#### Exclusive masking analysis

For the CPT, when the interaction effect of FAM was exclusively masked with that of LKM, the significant activations in the right thalamus and precuneus remained. Activation of the right MTG was also marginally significant (9 contiguous voxels). Alternatively, when the interaction effect of LKM was exclusively masked with that of FAM, no suprathreshold clusters remained.

For viewing happy pictures, when the interaction effect of FAM was exclusively masked with that of LKM, significant activation in the left insula remained. Alternatively, when the interaction effect of LKM was exclusively masked with that of FAM, significant activations in the left ventral ACC, right IFG, and precuneus remained.

For viewing sad pictures, when the interaction effect of FAM was exclusively masked with that of LKM, significant activation in the right IFG and left SFG remained. Alternatively, when the interaction effect of LKM was exclusively masked with that of FAM, significant activations in the left MFG and bilateral caudate remained.

To further support our claim that FAM and LKM do not share the same neural mechanism, the conjunction and exclusive masking of the main effect of state between FAM and LKM experts were performed and the results of which were reported separately in Additional Results S1.

#### Region-of-interest analysis

**Figure 3:**
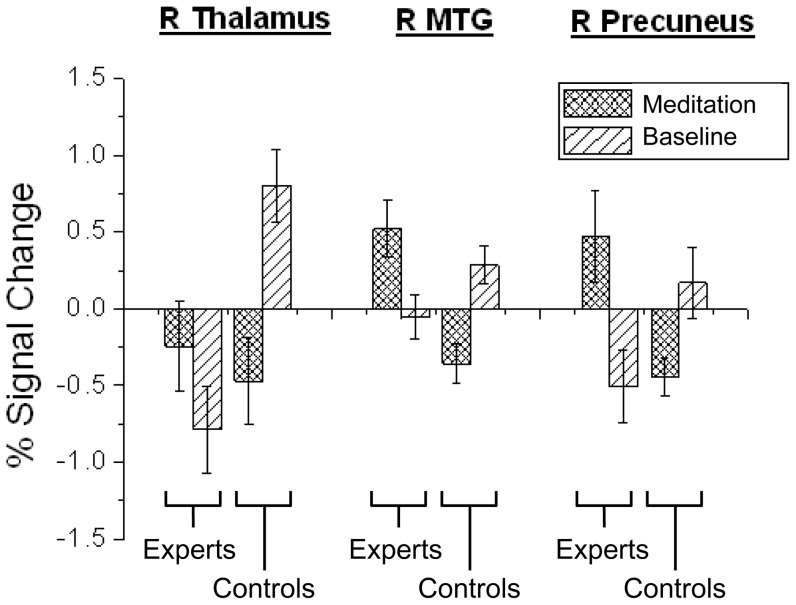
The percent signal change in brain regions showing significant interactions for CPT in FAM group. **Notes:** R: right; MTG: Middle temporal gyrus.

On the CPT, the complementary two-way factorial analysis using the ROI results obtained with the modified ROI masks replicated the interaction result for FAM (Figure 3). For the interaction in the right thalamus *F*(1,18) = 10.5, *p*<.005, both groups had similar activation during meditation, but FAM experts had significantly lower activity than FAM novices during baseline. For the interaction in the right MTG *F*(1,18) = 26.3, *p*<.001, FAM experts had significantly higher activity than FAM novices during meditation, but similar activation during baseline. For the interaction in the right precuneus *F*(1,18) = 9.6, *p*<.01, FAM experts had significantly higher activity than FAM novices during meditation, but similar activation during baseline.

#### Correlation analysis

For the CPT, there was only one significant correlation between performance and neural activity. For the FAM experts at baseline, activity in the right thalamus correlated positively with commission errors (*r* = .711, *p* = .03). For the LKM, there were no significant correlations between neural activity and performance.

For the EPT, there were two significant negative correlations between ratings of the IAPS pictures and neural activity. When the FAM experts were viewing happy pictures at baseline, activity in the left insula correlated negatively with both valence (*r* = −.618, *p* = .04) and arousal (*r* = −.655, *p* = .03).

## Discussion

This study investigated whether there was a dissociable effect on neural activity associated with attentional control and emotion processing between longterm FAM and LKM. Neural activity associated with performing on the experimental tasks was largely consistent with that reported in the literature (CPT [6], [31]; EPT [17], [32]). This observation provides reassuring evidence of the validity of the experimental paradigms used in this study.

Findings from conjunction and exclusive masking analyses offer evidence for a dissociable pattern of activation associated with FAM and LKM as evoked by the CPT and the EPT. This finding constitutes the first report that different forms of meditation have meditation-specific effects on neural activity, rather than a common neural mechanism. It clearly points to the idea that different forms of meditation practice create domain-specific plastic changes in neural activity [33]. Exclusive masking analyses gave further support to the prediction set forth in the *a priori* hypothesis that each form of meditation is associated with a dissociable pattern of neural activity during performing cognitive (CPT) and emotion (EPT) tasks. During the CPT, the FAM group (both experts and novices) showed a stronger state-by-group interaction of BOLD signals in attention-related regions. The LKM group did not show a significant state-by-group interaction. On the other hand, when processing affective pictures, both the FAM and the LKM groups showed distinct state-by-group interactions of BOLD signals in the emotion-processing neural system.

### Cognitive Performance

#### Focused-attention meditation

Behaviorally, the FAM experts made significantly fewer omission errors than the FAM novices during both the meditation and baseline states. This suggests that long-term practice of FAM is helpful with vigilance during tasks that require sustained attention. In terms of neural activity, the distinct patterns of BOLD signals associated with performing CPT in the FAM suggest that practicing FAM may be associated with enhancing attention-specific brain areas. We can differentiate FAM experts and novices by activity in the right thalamus, right MTG, and right precuneus, as shown by the similar results from the *post hoc t*-tests and ROI analyses. Experts had weaker BOLD signals in the right thalamus than the novices during baseline. Furthermore, activity of the right thalamus was positively associated with the rates of commission errors in experts during baseline. Previous research has suggested that the right thalamus is involved in the attention processes measured by CPT and similar paradigms [14]–[16]. Furthermore, lower thalamic activity appears to be associated with high arousal, as well as a subjective feeling of needing less mental load to complete attention tasks [34]. Hence, the weaker BOLD signals in the right thalamus among the experts is consistent with the notion that longterm practice of FAM may bring about a clear and unwarvering mind to increase attentional stability and reduce task effort [6], [35].

Experts also had stronger BOLD signals in the right MTG during meditation, compared to the novices. Researchers have suggested that the temporal cortex is associated with maintaining attention [36] and controlling bottom-up, stimulus-driven attention, especially during tasks like the CPT that involve contingencies (i.e., responding to randomly presented target stimulus; [37]. Indeed, a previous study found that activity in the MTG was negatively correlated with reaction time during CPT [15]. Thus, we interpreted the stronger BOLD signals in the MTG during meditation as implying that FAM facilitates accurate and efficient responses to target stimuli.

Experts had stronger BOLD signals in the right precuneus during meditation, compared to the novices. In a sustained attention study, healthy volunteers demonstrated reduced precuneus activity during later task trials, suggesting that the reduced precuneus activity could serve as an index of task familiarity [38]. This observation may reflect how alertness decreases upon prolonged exposure—habituation. This is consistent with the observation that people undergo a profound deactivation in the precuneus and the adjacent posteromedial cortex during altered states of consciousness, such as sleep [39]. Therefore, increased BOLD signals in the right precuneus of the experts during meditation may help them sustain their attention without being affected by the reduced novelty of the CPT. On the other hand, it seems there was a trend that they made fewer commission errors than the FAM novices during the meditation state but not during the baseline state. Hence, among the FAM experts, there may be a trait-like effect on controlling for omission errors but a state-like effect on inhibiting commission errors.

#### Loving-kindness meditation

During the CPT, no significant findings on state-by-group interaction in any neural regions were observed. This suggests that longterm LKM may not be associated with change in attention-related regions.

### Affective Processing

The effects of FAM and LKM on brain activity when processing affective pictures were very different. The results of the conjunction and exclusive masking analyses corroborate the hypothesized dissociable neural activation pattern associated with the processing of affective photos between the FAM and LKM experts.

#### Focused-attention meditation

Consistent with previous findings, our findings suggest that the impact of longterm FAM on brain activity extends from attentional control to emotion processing [5]. Attention-related meditation might involve a more elaborative processing of the features of emotional stimuli, leading to higher activity in the ventral neural system [40]. Our findings add to the current literature by showing the impact of FAM on emotion processing, adding to the previous evidence for the cognitive impact of attention-related meditation [2], [41], [42].

While viewing happy pictures, experts had significantly stronger BOLD signals in the left (anterior) insula during meditation versus baseline. Activity in the left anterior insula is related to the interaction between arousal and valence when viewing affective pictures [43]. In this study, our FAM experts, relative to the novices, gave marginally higher arousal ratings to the happy pictures (*p* = .08). Furthermore, their arousal and valence ratings were negatively correlated with activity in the left insula. These findings are unexpected because literature has suggested a positive relationship between insular activity and emotion intensity [43], [44]. We thought that the insular activity in the FAM expert may relate to the possiblity that these experts, different from the novices who do not usually require to regulate the positive emotion associated with viewing happy pictures, might have attempted to suppress the affective impact of these positive pictures, for arriving at the state of tranquillity. Hence, there was greater insular activity but lower arousal and valence ratings for these FAM experts.

There were two findings worth noting for viewing sad pictures. The experts had significantly stronger BOLD signals in the left SFG, both in meditation and at baseline, compared with the novices' baseline state. Furthermore, novices had stronger BOLD signals in the right IFG during meditation versus baseline. FAM is believed to be associated with remarkably lower emotional reactivity, which is important for maintaining emotional stability and a focused state [2]. Activity in the dorsolateral frontal regions is associated with monitoring and attentional orienting in FAM (meditation vs. baseline state) [6]. Therefore, when the experts and novices were in a focused meditative state, they may have recruited these two regions more than the novices did during baseline in order to maintain emotional stability. We suggest that the lower emotional reactivity of experts may come from—at least partly—a generalized effect of longterm practice, even when the form of meditation practice does not emphasize emotion processing.

Despite the absence of behavioral difference on valence and arousal ratings between the experts and novices, their different neural activities when performing the EPT could be interpreted as reflecting the different neural mechanisms and pathways for processing affective stimuli adopted by these two groups. This speculation was supported by previous literature on sex-related difference in neural processing of affective stimuli, which also reported similar levels of behavioral outputs between men and women [21], [45].

#### Loving-kindness meditation

Our findings add to the base of evidence from the recent surge of LKM research and practice. LKM cultivates a generalized feeling of love and compassion towards all humankind and living creatures without causing significant distress to practitioners.

While viewing happy pictures, activity in the left ventral ACC showed both state difference (meditation>baseline) within the experts and group difference (experts>novices). Activity in the right IFG showed only group difference in activation level (experts>novices) during meditation. Also, experts had stronger BOLD signals in the right precuneus during meditation versus baseline.

The left ventral ACC in the ventral neural system is important for identifying the emotional value of stimuli and producing the corresponding affective state. In contrast, the right IFG in the dorsal affective processing system is important for regulating emotional responses [40]. Cavanna and Trimble (2006) suggest that the precuneus forms a classic network with the right PFC through the ACC, which is implicated in episodic memory retrieval and self-referential processing. Therefore, the cultivation of love and kindness in the practice of LKM may allow experts to be more capable of sharing the positive emotions of others by feeling the happiness of others as their own and further wishing for others' happiness [40]. It seems that LKM practice is associated with activity in these emotion-processing regions, which may have an impact on emotion regulation and the subsequent production of positive emotions.

While viewing sad pictures, the experts, relative to the novices, showed stronger BOLD signals in the left caudate and MFG during meditation. On the other hand, novices showed stronger BOLD signals in the right caudate during baseline than meditation state. Previous literature has reported that the striatum (which includes the caudate) was related to processing positive affect, such as reward. Some recent literature, however, has shown that the caudate is also involved in aversive affective processing, such as of processing negative words [46] and unpleasant pictures [47]. Activity in the left caudate seems to be associated with the arousal level of emotions [48]. Furthermore, in the framework of appraisal theory, activity of the dorsolateral prefrontal cortex is indicated in voluntary and effortful regulation of emotional responses [40]. Since activity of the left caudate and MFG of LKM experts were negatively correlated with the arousal level of the sad pictures seen in this study, taken into the consideration of the roles played by the caudate and the lateral prefrontal cortex as discussed above, we suggest that LKM might be related to higher emotion reactivity in conjunction with more efficient voluntary emotion regulation, which would be consistent with the idea that emotion regulation helps distinguish empathy from emotional contagion and distress [49]. Since LKM cultivates the feelings of love, kindness, and compassion towards affective stimuli, it might automatically engage purposive emotion regulation. These strategies could be cognitive reappraisal, distraction, or expressive suppression [50]–[52]; future studies are needed to determine which strategy (or strategies) is used.

According to a recent study of the neural correlates of exposure to death-related thoughts, activity in the right caudate increased strongly when subjects answered death-related questions [53]. It was speculated that the right caudate is involved in the automatic psychological defense against mortality threat because of its role in habitual behaviors [54]. Approximately 85% of our sad pictures are related to the sorrow of death (e.g., scenes of graves, dying patients or animals, and war), which may explain the strong right caudate activity in the novices when they were viewing the sad pictures in the baseline state. The basal ganglia, including the caudate and the putamen, are involved in the production of negative emotions [55], [56]. An increase in negative affect has been associated with increases in right-sided activation in the orbitofrontal and dorsolateral prefrontal cortices [57], regions that are closely connected to the right caudate [58]. According to literature, people with a strong right-sided activation appear to be slower in recovering from negative affect or stress than people with a strong left-sided activation [57].

### Limitations

A number of limitations should be discussed. First, the participants were a convenience sample of Chinese meditation practitioners in a Buddhist network in Hong Kong. This subculture and ethnicity may affect the psychological and physiological correlates of meditation. However, it is hard to know anything about ethnic differences because most previous studies used samples with mixed ethnicities [4], [59]. Caution is therefore warranted before generalizing these findings to other populations. Also, all the participants were men, which limits the generalization of the findings to female populations.

Due to time constraints, we were unable to directly measure participants' attention to and processing of the stimuli while they performed the task. This makes it more difficult to interpret the findings on emotion processing: some participants might be more adept at reducing emotional reactivity by redirecting their attention away from emotional stimuli consciously or automatically without awareness [60]. In the postexperiment debriefing session, we asked participants whether they were able to maintain the clarity and stability of their mental states while performing the two experimental tasks. Future studies should consider incorporating objective measures of visual attentiveness to the experimental stimuli, such as eletrooculography and infrared video cameras.

### Conclusions

These limitations notwithstanding, this study helps advance neuroscientific research on meditation. Our findings suggest that FAM and LKM have dissociable effects on the neural activity associated with attention and emotion processing. Specifically, we demonstrated that the practice of FAM was associated with expertise-related behavioral improvements and neural activation differences in attention task performance. However, the effect of state LKM meditation did not carry over to attention task performance. On the other hand, both FAM and LKM practice appeared to affect the neural responses to affective pictures. For viewing sad faces, the regions activated for FAM practitioners were consistent with attention-related processing; whereas responses of LKM experts to sad pictures were more in line with differentiating emotional contagion from compassion/emotional regulation processes. These observations contribute to the literature on neuroplasticity by adding evidence that practice is associated with specific effects on brain activity. Meditation does influence emotion processing, regardless of whether the practice focuses on cognition (*ānāpānasati*) or emotion (*mettā*). Finally, the neural pathways underlying emotion processing associated with LKM are likely to be different from those associated with FAM.

## Supporting Information

Figure S1 **BOLD signals associated with performing the Continuous Performance Test (CPT) and Emotion Processing Task (EPT) by novices at the baseline state.** **Notes**: (a) Neural activity of all novices performing the CPT (experimental>control condition) at a resting state (*p*<.001, k = 10); (b) Neural activity of all novices while viewing happy (*p*<.001, k = 10), and (c) sad picture (*p*<.005*∧*, k = 10) (comparing with viewing neutral pictures) in the EPT during the resting state. L: left, R: Right, STG: Superior Temporal Gyrus, MTG: Middle Temporal Gyrus, ITG: Inferior Temporal Gyrus, MOG: Middle Occipital Gyrus, SFG: Superior Frontal Gyrus, MFG: Middle Frontal Gyrus, ACC: Anterior Cingulate Cortex, PCC: Posterior Cingulate Cortex, IPL: Inferior Parietal Lobe. *∧ The threshold was relaxed because no suprathreshold clusters were detected at p<.001.*
(DOC)

Figure S2 **The whole-brain results of significant 3-way interaction effects (state by group by form-of-meditation) for each condition were shown below.** A threshold of *p*<0.001, k>10 was used, which is the same as that used for the 2-way analyses in the main text. The differences between these 3-way results and the 2-way results reported in the main text were likely due to insufficient power because of a small sample size. **Notes**: (a) CPT: Continuous Performing Task; (b) and (c) EPT: Emotion Processing Test. L: left, R: Right, MFG: Middle Frontal Gyrus, IFG: Inferior Frontal Gyrus, ACC: Anterior Cingulate Cortex.
(DOC)

Table S1 **Main effect of state of the whole-brain voxel-wise ANOVA for different types of meditation in the three tasks.**
(DOC)

Additional Results S1. (DOC)
