# Supplementary material for: Distinct Neural Activity Associated with Focused-Attention Meditation and Loving-Kindness Meditation
Source: PLoS One. 2012 Aug 15;7(8):e40054. doi: 10.1371/journal.pone.0040054 (PMC3419705; doi:10.1371/journal.pone.0040054)
Supplement: Figure S1 — BOLD signals associated with performing the Continuous Performance Test (CPT) and Emotion Processing Task (EPT) by novices at the baseline state. Notes: (a) Neural activity of all novices performing the CPT (experimental>control condition) at a resting state (p<.001, k = 10); (b) Neural activity of all novices while viewing happy (p<.001, k = 10), and (c) sad picture (p<.005∧, k = 10) (comparing with viewing neutral pictures) in the EPT during the resting state. L: left, R: Right, STG: Superior Temporal Gyrus, MTG: Middle Temporal Gyrus, ITG: Inferior Temporal Gyrus, MOG: Middle Occipital Gyrus, SFG: Superior Frontal Gyrus, MFG: Middle Frontal Gyrus, ACC: Anterior Cingulate Cortex, PCC: Posterior Cingulate Cortex, IPL: Inferior Parietal Lobe. ∧ The threshold was relaxed because no suprathreshold clusters were detected at p<.001. (DOC) [file pone.0040054.s001.doc]

**Figure S1:** BOLD signals associated with performing the Continuous Performance Test (CPT) and Emotion Processing Task (EPT) by novices at the baseline state.

**
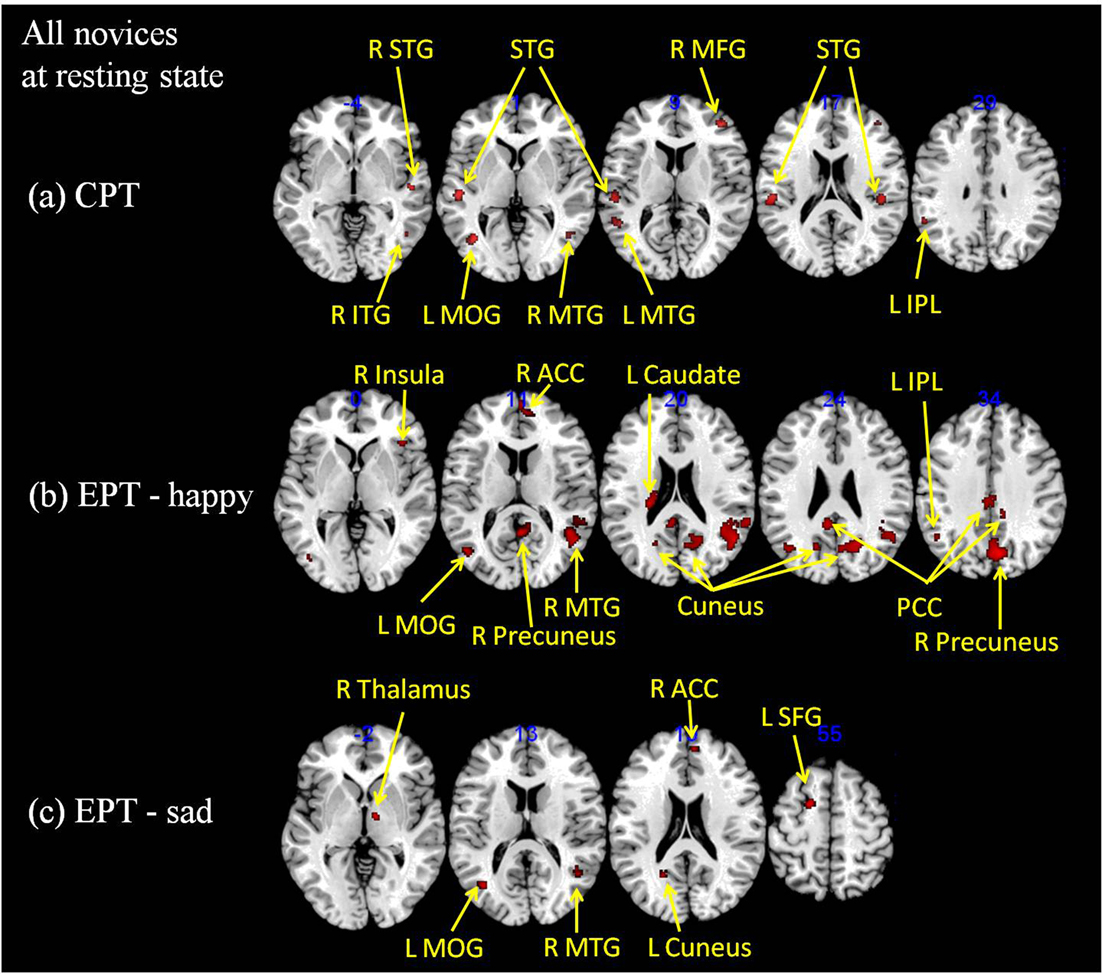
**

**Note**: (a) Neural activity of all novices performing the CPT (experimental > control condition) at a resting state (*p*<.001, k=10); (b) Neural activity of all novices while viewing happy (*p*<.001, k=10), and (c) sad picture (*p*<.005*^*, k=10) (comparing with viewing neutral pictures) in the EPT during the resting state.

L: left, R: Right, STG: Superior Temporal Gyrus, MTG: Middle Temporal Gyrus, ITG: Inferior Temporal Gyrus, MOG: Middle Occipital Gyrus, SFG: Superior Frontal Gyrus, MFG: Middle Frontal Gyrus, ACC: Anterior Cingulate Cortex, PCC: Posterior Cingulate Cortex, IPL: Inferior Parietal Lobe.

*^ The threshold was relaxed because no suprathreshold clusters were detected at p<.001.*
