# Supplementary material for: Distinct Neural Activity Associated with Focused-Attention Meditation and Loving-Kindness Meditation
Source: PLoS One. 2012 Aug 15;7(8):e40054. doi: 10.1371/journal.pone.0040054 (PMC3419705; doi:10.1371/journal.pone.0040054)
Supplement: Figure S2 — The whole-brain results of significant 3-way interaction effects (state by group by form-of-meditation) for each condition were shown below. A threshold of p<0.001, k>10 was used, which is the same as that used for the 2-way analyses in the main text. The differences between these 3-way results and the 2-way results reported in the main text were likely due to insufficient power because of a small sample size. Notes: (a) CPT: Continuous Performing Task; (b) and (c) EPT: Emotion Processing Test. L: left, R: Right, MFG: Middle Frontal Gyrus, IFG: Inferior Frontal Gyrus, ACC: Anterior Cingulate Cortex. (DOC) [file pone.0040054.s002.doc]

**Figure S2.**

The whole-brain results of significant 3-way interaction effects (state by group by form-of-meditation) for each condition were shown below. A threshold of *p* < 0.001, k > 10 was used, which is the same as that used for the 2-way analyses in the main text. The differences between these 3-way results and the 2-way results reported in the main text were likely due to insufficient power because of a small sample size.

**
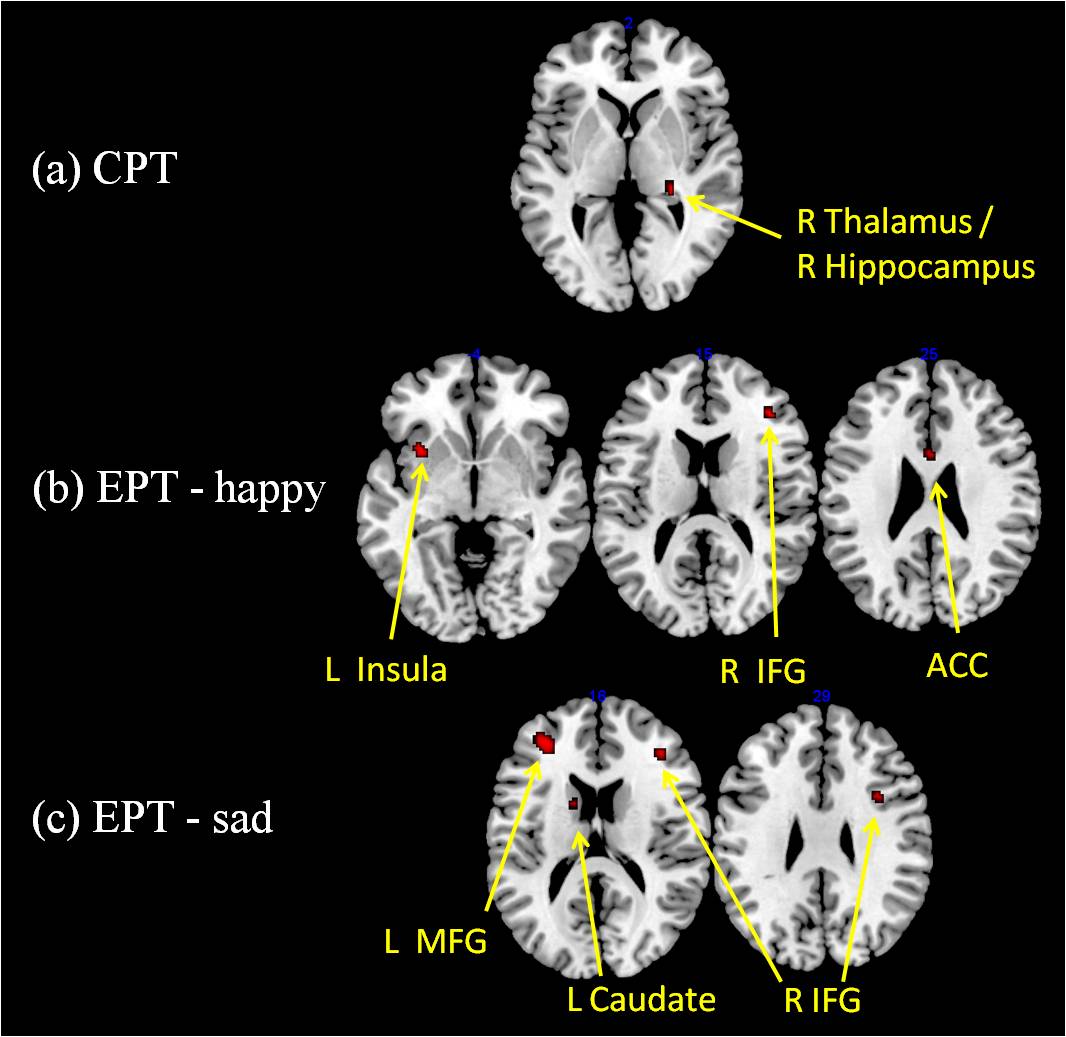
**

**Note**: (a) CPT: Continuous Performing Task; (b) and (c) EPT: Emotion Processing Test

L: left, R: Right, MFG: Middle Frontal Gyrus, IFG: Inferior Frontal Gyrus, ACC: Anterior Cingulate Cortex
