# Supplementary material for: Distinct Neural Activity Associated with Focused-Attention Meditation and Loving-Kindness Meditation
Source: PLoS One. 2012 Aug 15;7(8):e40054. doi: 10.1371/journal.pone.0040054 (PMC3419705; doi:10.1371/journal.pone.0040054)
Supplement: Table S1 — Main effect of state of the whole-brain voxel-wise ANOVA for different types of meditation in the three tasks. (DOC) [file pone.0040054.s003.doc]

**Table S1. Main effect of state of the whole-brain voxel-wise ANOVA for different types of meditation in the three tasks**

| Task | Meditation | Brain region of significant main effect of state | Coordinates | | | F value | Cluster size | *t* value | |
| --- | --- | --- | --- | --- | --- | --- | --- | --- | --- |
|  |  |  | x | y | z |  |  | Experts | Novices |
|  |  |  |  |  |  |  |  |  |  |
| (a) CPT | FAM | *no suprathreshold voxels* |  |  |  |  |  |  |  |
|  |  |  |  |  |  |  |  |  |  |
|  | LKM | L Putamen | -26 | 8 | 8 | 17.09 | 51 | 3.20 ^a^ | 4.92 ^a^ |
|  |  |  | -22 | 0 | -6 | 14.64 |  |  |  |
|  |  |  | -26 | 6 | -2 | 14.04 |  |  |  |
|  |  | R Inferior Frontal Gyrus | 28 | 20 | 30 | 15.89 | 30 | X | 3.43 ^b^ |
|  |  |  |  |  |  |  |  |  |  |
| (b) EPT-happy | FAM | R Postcentral Gyrus | 28 | -24 | 46 | 16.43 | 13 | 3.67 ^b^ | 3.41 ^b^ |
|  |  | R Inferior Temporal Gyrus | 50 | -68 | -4 | 15.98 | 15 | X | 3.24 ^b^ |
|  |  | R Fusiform Gyrus | 40 | -68 | -20 | 15.86 | 11 | X | 3.63 ^b^ |
|  |  | Middle Cingulate Cortex | 2 | 8 | 32 | 15.68 | 12 | 3.51 ^b^ | X |
|  |  |  |  |  |  |  |  |  |  |
|  | LKM | L Medial Frontal Gyrus | -14 | 42 | 26 | 17.42 | 15 | 3.50 ^b^ | 2.95 ^b^ |
|  |  | R Superior Frontal Gyrus | 18 | 46 | 30 | 16.46 | 11 | 3.64 ^b^ | 3.54 ^b^ |
|  |  |  |  |  |  |  |  |  |  |
| (c) EPT-sad | FAM | R Hippocampus | 18 | -26 | -10 | 21.03 | 16 | 2.81 ^b^ | 4.13 ^b^ |
|  |  | L Middle Occipital Gyrus | -40 | -78 | 0 | 20.84 | 101 | 3.00 ^b^ | 3.76 ^b^ |
|  |  | R Superior Occipital Gyrus | 18 | -86 | 30 | 20.44 | 42 | 2.71 ^b^ | 4.64 ^b^ |
|  |  | L Calcarine | -22 | -70 | 8 | 17.83 | 29 | 4.06 ^b^ | 3.55 ^b^ |
|  |  |  | -22 | -80 | 10 | 14.78 |  |  |  |
|  |  | R Cuneus extends to R Superior Occipital Gyrus | 20 | -86 | 12 | 17.13 | 56 | X | 4.66 ^b^ |
|  |  |  | 34 | -76 | 8 | 16.13 |  |  |  |
|  |  |  | 32 | -86 | 12 | 14.88 |  |  |  |
|  |  | L Calcarine | -8 | -82 | 10 | 15.21 | 28 | X | 3.60 ^b^ |
|  |  |  |  |  |  |  |  |  |  |
|  | LKM | L Superior Frontal Gyrus | -10 | 30 | 48 | 19.67 | 17 | 3.99 ^b^ | 3.66 ^b^ |
|  |  |  | -16 | 24 | 48 | 18.4 |  |  |  |
|  |  |  |  |  |  |  |  |  |  |

L = Left, R = Right, X = non-significant

^a^ meditation < baseline, ^b^ meditation > baseline
