# Supplementary material for: Distinct Neural Activity Associated with Focused-Attention Meditation and Loving-Kindness Meditation
Source: PLoS One. 2012 Aug 15;7(8):e40054. doi: 10.1371/journal.pone.0040054 (PMC3419705; doi:10.1371/journal.pone.0040054)
Supplement: Additional Results S1. — (DOC) [file pone.0040054.s004.doc]

**Additional results S1:**

We have performed the conjunction analysis of the main effect of the state between FAM and LKM experts in each group. Using the procedures and thresholding methods as described in “fMRI data analysis” in the manuscript, we observed that there were not any commonly activated regions for the state effect (meditation vs. baseline) for all three tasks. Therefore, the two types of meditation do not share the same neural mechanism.

The exclusive masking of the main effect of state between FAM and LKM across the expert groups showed similar results compared to that across both the expert and novice groups.

For the CPT, when the main effect of state of FAM was exclusively masked with that of LKM, a significant activation of the right middle/superior temporal gyrus was observed. This activity was also found when interaction effects were used in the manuscript (despite marginal significance). Alternatively, when the main effect of state of LKM was exclusively masked with that of FAM, no suprathreshold clusters were resulted. This is the same as the exclusive masking result when interaction effects were used.

For viewing happy pictures, when the main effect of state of FAM was exclusively masked with that of LKM, significant activations in the left precentral and postcentral gyri and middle cingulate cortex were detected. These findings are different from that using the interaction effects in the manuscript. Alternatively, when the main effect of state of LKM was exclusively masked with that of FAM, significant activations in the left ventral ACC, left IFG, and left precuneus were observed. These results are highly similar to that using the interaction effect.

For viewing sad pictures, when the main effect of state of FAM was exclusively masked with that of LKM, significant activations in the right caudate, left middle/inferior frontal gyrus and left putamen were observed. These results are different from that using the interaction effects. Alternatively, when the main effect of state of LKM was exclusively masked with that of FAM, a significant activation in the left MFG was detected. This activity was also observed when interaction effects were used.
